# Vascular-targeted therapies for Duchenne muscular dystrophy

**DOI:** 10.1186/2044-5040-3-9

**Published:** 2013-04-23

**Authors:** James P Ennen, Mayank Verma, Atsushi Asakura

**Affiliations:** 1 Stem Cell Institute, University of Minnesota Medical School, McGuire Translational Research Facility, Room 4-220, 2001 6th Street SE, Minneapolis, MN 55455, USA; 2 Paul and Shelia Wellstone Muscular Dystrophy Center, University of Minnesota Medical School, Wallin Medical Biosciences Building, 2101 6th Steet SE, Minneapolis, MN 55455, USA; 3 Department of Neurology, University of Minnesota Medical School, Phillips Wangensteen Building, 420 Delaware Street SE, Minneapolis, MN 55455, USA; 4 University of Minnesota Medical Scientist Training Program (MD/PhD), Mayo Building, Room B-681, 420 Delaware Street SE, Minneapolis, MN 55455, USA

**Keywords:** Duchenne muscular dystrophy, VEGF, Flt-1, Flk-1, Nitric oxide, PDE5 inhibitor, ACE inhibitor, Satellite cell, Muscle regeneration, Myofiber damage

## Abstract

Duchenne muscular dystrophy (DMD) is the most common muscular dystrophy and an X-linked recessive, progressive muscle wasting disease caused by the absence of a functional dystrophin protein. Dystrophin has a structural role as a cytoskeletal stabilization protein and protects cells against contraction-induced damage. Dystrophin also serves a signaling role through mechanotransduction of forces and localization of neuronal nitric oxide synthase (nNOS), which produces nitric oxide (NO) to facilitate vasorelaxation. In DMD, the signaling defects produce inadequate tissue perfusion caused by functional ischemia due to a diminished ability to respond to shear stress induced endothelium-dependent dilation. Additionally, the structural defects seen in DMD render myocytes with an increased susceptibility to mechanical stress. The combination of both defects is necessary to generate myocyte damage, which induces successive rounds of myofiber degeneration and regeneration, loss of calcium homeostasis, chronic inflammatory response, fibrosis, and myonecrosis. In individuals with DMD, these processes inevitably cause loss of ambulation shortly after the first decade and an abbreviated life with death in the third or fourth decade due to cardio-respiratory anomalies. There is no known cure for DMD, and although the culpable gene has been identified for more than twenty years, research on treatments has produced few clinically relevant results. Several recent studies on novel DMD therapeutics are vascular targeted and focused on attenuating the inherent functional ischemia. One approach improves vasorelaxation capacity through pharmaceutical inhibition of either phosphodiesterase 5 (PDE5) or angiotensin-converting enzyme (ACE). Another approach increases the density of the underlying vascular network by inducing angiogenesis, and this has been accomplished through either direct delivery of vascular endothelial growth factor (VEGF) or by downregulating the VEGF decoy-receptor type 1 (VEGFR-1 or Flt-1). The pro-angiogenic approaches also seem to be pro-myogenic and could resolve the age-related decline in satellite cell (SC) quantity seen in *mdx* models through expansion of the SC juxtavascular niche. Here we review these four vascular targeted treatment strategies for DMD and discuss mechanisms, proof of concept, and the potential for clinical relevance associated with each therapy.

## Review

Duchenne muscular dystrophy (DMD) is an X-linked recessive, progressive muscle wasting disease caused by mutations in the *DMD* gene that lead to absence of a functional dystrophin protein [1,2]. Both fatal and devastating, DMD is the most common muscular dystrophy seen in children and has an annual incidence affecting one in every 3600–6000 newborn males [3]. Normally, dystrophin serves as the bridge in the dystrophin-associated glycoprotein complex (DAPC), connecting the cytoskeleton, via attachments to subsarcolemmal F-actin, to the extracellular matrix through an association with plasma membrane bound β-dystroglycan [4]. In the DAPC, dystrophin has a structural role as a cytoskeletal stabilization protein and protects cells against contraction-induced damage. Dystrophin also serves signaling roles, including mechanotransduction of forces and localization of signaling proteins, such as neuronal nitric oxide synthase (nNOS), which synthesizes nitric oxide (NO) to facilitate vasorelaxation [5-7]. Without dystrophin, the DAPC cannot completely assemble, and the supportive link between the cytoskeleton and the extracellular matrix becomes destabilized [8]. Despite normal development, the membrane in dystrophin-deficient cells is easily damaged. Membrane microlesions facilitate an influx of calcium ions, which activate proteases to begin auto-digestion of the musculature sarcoplasm [9-11]. Macrophages later arrive at the tissue to remove cellular debris, and satellite cells (SCs) are activated and proliferate to induce myofiber regeneration. This causes successive rounds of myofiber degeneration and regeneration that is exacerbated by continual membrane damage and ensuing myonecrosis. In addition, cytokines released in the process of myonecrosis recruit inflammatory cells, which release inflammatory cytokines to activate fibroblasts that lay down extracellular matrix proteins and lead to fibrosis [12]. Skeletal muscle regenerative capacity later diminishes with advancing age and decreasing numbers of SCs, and muscle tissue is steadily replaced by adipose and connective tissues [13].

**Figure 1:**
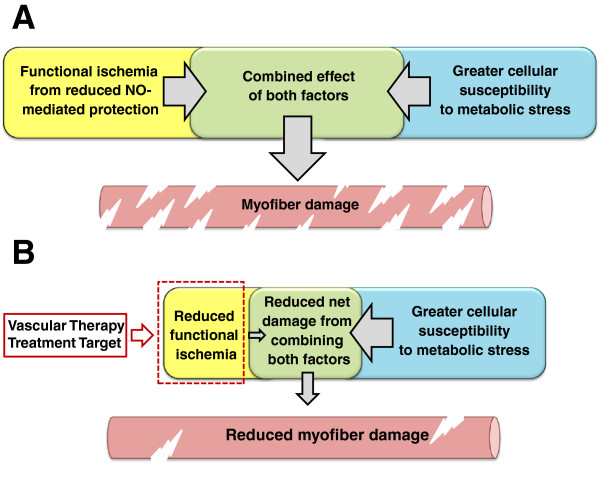
**The two-hit hypothesis for myocyte damage and the proposed outcome of functional ischemia attenuation in Duchenne muscular dystrophy (DMD).** (**A**) The combined effects from functional ischemia due to reduced nitric oxide (NO)-mediated protection and greater cellular susceptibility to metabolic stress are necessary to produce the myofiber damage observed in DMD [17]. (**B**) Attenuating functional ischemia by administering a vascular targeted treatment can reduce the net-combined effect of both two-hit factors and consequently curtail myofiber damage.

The previously described cellular events manifest themselves clinically in a devastating and progressive manner. Despite continuous contractions by the myocardium, the skeletal muscles deteriorate first in individuals with DMD, and most permanently lose ambulatory abilities shortly after the first decade [14]. Myocardial problems present later, and clinically relevant cardiomyopathy is seen in 90% of patients over 18 years old, namely due to the onset of cardiac fibrosis in addition to rhythm and conduction abnormalities [14]. Respiratory problems are also inevitable due to muscle wasting in the diaphragm and the onset of scoliosis [14]. Even with improvements in treatment, notably multidisciplinary care, the combined cardio-respiratory anomalies mean that most individuals with DMD die in their third or fourth decade of life [15,16]. Despite knowledge of the responsible gene for over twenty years, a DMD cure remains to be found, and research on treatments has produced few clinically relevant results. Current treatment options, such as corticosteroid administration, physical therapy, nocturnal ventilation, and surgical interventions aim for symptomatic management and have been shown to improve lifespan and quality of life [16]. The clinical utility and feasibility of gene therapy and cell therapy remain to be elucidated, and other treatment areas must be sought. Our current, more holistic understanding of DMD pathogenesis, especially with more recent knowledge of the vascular role of dystrophin, implies that vascular-targeted therapies are strong candidates for future investigation. Specifically, attenuating functional ischemia could reduce myocyte damage, increase tissue perfusion, reduce cardiac workload, and prevent cardiac and skeletal muscle remodeling (Figure 1B). This review will focus on vascular-targeted treatment avenues aimed at either improving vasorelaxation capacity or increasing the underlying vascular density in order to reduce the functional ischemia and improve the DMD phenotype.

### Defect of nitric oxide-mediated vasodilation contributes to Duchenne muscular dystrophy phenotype

The DMD pathogenesis is partially explained by the lack of the signaling role of dystrophin, which normally localizes nNOS to the sarcolemma through binding to the C-terminal region of dystrophin [6]. The nNOS is responsible for NO production to facilitate smooth muscle vasodilation in response to increased metabolic demands. During muscle contraction, NO-mediated vasodilation is important to help offset the α-adrenergic vasoconstriction in response to sympathetic activation, which optimizes muscle perfusion [18]. This functional response is intact in healthy children, but in children with DMD the sympathetic vasoconstriction in skeletal muscle is unopposed due to lack of NO-mediated vasodilation [18].

The nNOS is absent from the sarcolemma and is greatly downregulated in the cytoplasm of dystrophin-deficient muscle, which results in muscle vasoconstriction and abnormal blood flow during skeletal muscle contraction [6,18,19]. Specifically, loss of dystrophin in the smooth muscle results in a decreased capacity of the vasculature to respond to shear stress induced endothelium-dependent dilation, probably related to the signaling defects seen in both force transduction and inadequate NO production [19]. Furthermore, shear stress at the endothelial cell surface is a known catalyst for angiogenesis [20-23], so new blood vessel formation could be downregulated and mismatched to metabolic need in the absence of dystrophin due to defects in mechanotransduction. Lack of the signaling and structural roles of dystrophin in DMD pathogenesis have led to a two-hit hypothesis, whereby the combination of functional ischemia due to reduced capacity to benefit from NO-mediated protection and an increased susceptibility to metabolic stress are both required to cause myocyte damage (Figure 1A) [17]. So although not completely culpable for the observed pathogenesis, impaired vascular functioning seems to be both inherent to DMD and an accelerant to tissue damage in the skeletal and cardiac muscles.

Recent studies suggest that the two-hit hypothesis should migrate away from simply observational speculation towards a more widely accepted, evidence-based DMD pathogenic theory. One functional study using model DMD *mdx* mice showed quantitative evidence supporting the two-hit hypothesis, where inhibition of NO/EDHF (EDHF is endothelium-derived hyperpolarizing factor, another vasodilator) alone in wild-type mice caused similar functional ischemia (one-hit) to that seen in *mdx* mice [24]. But, the forced functional ischemia alone in the wild-type mice did not induce similar levels of muscle cell death seen in *mdx* mice [24]. Two-hits, consisting of severe ischemia and strenuous tetanic stimuli, were necessary to produce the same contraction-dependent myofiber damage in wild-type mice to that of *mdx* mice [24]. So, *mdx* myofibers exhibit enhanced vulnerability to metabolic and mechanical stress independently of the altered vasodilatory response, yet the combinatory effect of *both* factors (two-hits) is necessary to mediate the cell death numbers seen in *mdx* myofibers. This could also explain why nNOS knockout mice, a one-hit model, do not develop muscular dystrophy, yet myocardial specific nNOS expression prevents cardiomyopathy in *mdx* mice by increasing the capacity to benefit from NO-mediated protection [25,26]. Interestingly, *mdx* mice that only express dystrophin in smooth muscle (*SMTg/mdx*), which has a prominent role in regulation of vascular tone and blood flow, have an intermediate phenotype between wild-type and *mdx* mice [27]. The *SMTg/mdx* mice showed some, albeit not total, recovery of the NO-dependent vasorelaxation mechanism in active skeletal muscle [27]. In the *SMTg/mdx* model, lack of dystrophin at the myofiber level and the consequent lack of complete force mechanotransduction in response to functional demands were perhaps partially responsible for the insufficient phenotypic recovery. These data overwhelmingly reveal the important role the vasculature plays in DMD pathogenesis and highlight a novel arena for therapeutic intervention.

### Improved vasorelaxation capacity

#### Angiotensin-converting enzyme inhibitors

The renin-angiotensin-aldosterone system plays a vital role in regulating both systemic vascular resistance and total blood volume, which together impact arterial pressure and myocardial function. A key component is the angiotensin-converting enzyme (ACE), which transforms the peptide hormone angiotensin I into angiotensin II; circulating angiotensin II then stimulates vascular smooth muscle contraction, increasing vascular resistance and arterial pressure. Angiotensin II also induces the release of aldosterone, which increases sodium and water retention, and vasopressin, which increases water retention. Preventing angiotensin II production through pharmacological ACE inhibition has been shown to reduce high blood pressure and cardiac workload through enhanced vasorelaxation and prevention of downstream hormone release, and ACE inhibitors (ACEIs) are currently used to treat congestive heart failure and hypertension [28-30]. As such, improving cardiac function and enhancing systemic vasorelaxation capacity through ACE inhibition in DMD patients could have prophylactic benefit by mitigating the functional ischemia and consequently diminishing myonecrosis. In *mdx* mice, the ACEI captopril administered over an 8-week period and prior to the onset of cardiomyopathy was shown to reduce cardiac afterload, increase myocardial contractility, and improve cardiac hemodynamics compared to *mdx* control mice [31]. In clinical studies, administration of the ACEI perindopril has shown that early treatment in 9.5- to 13-year-old DMD patients with normal cardiac functioning (as measured by normal left ventricular ejection fraction or LVEF), is capable of delaying both the onset and progression of left ventricular dysfunction and significantly lowering mortality rates compared to patients starting treatment 3 years later [32,33]. Results are less clear in DMD cases involving established cardiomyopathy where administration of the ACEI enalapril showed functional normalization in just 43% of cases, but this positive functional effect was maintained by most subjects for up to four years [34].

Combination therapy using ACEIs and β-adrenergic receptor antagonists or β-blockers (BBs) has also been investigated for use in DMD treatment. Catecholamines increase heart rate and myocardial contractility through the β-adrenergic receptors, and thus targeted BBs cause the heart to beat slower and with less force and are typically given to patients with arrhythmia or disordered automaticity. One study that utilized a combination of BBs and ACEIs in DMD patients with established cardiomyopathy found a positive effect on long-term survival, especially for individuals that showed no overt symptoms of heart failure despite documented left ventricular dysfunction [35]. A more recent study also investigated DMD patients with established cardiomyopathy but broke treatment groups down into ACEI (lisinopril) alone or ACEI plus BB (metoprolol) [36]. Both treatment groups displayed improvements in cardiac function compared to pre-therapy measurements, but no significant difference in cardiac function was seen between groups [36]. Future studies should address treatment using ACEI alone or ACEI plus BB in DMD cases where cardiomyopathy has not been fully established to assess the potential for prophylactic benefit as this could definitively rule out the need for a BB. Additionally, with regard to the two-hit hypothesis, studies that address functional ischemia attenuation to mitigate myonecrosis through enhanced tissue perfusion by ACEI-mediated vasorelaxation have not yet been performed.

Another therapeutic strategy that targets the renin-angiotensin-aldosterone system to improve vasorelaxation capacity utilizes the antihypertensive drug losartan, which is an angiotensin II type I receptor antagonist or angiotensin receptor blocker (ARB). Long-term administration of losartan in *mdx* mice showed improvements in myocardial function, but not skeletal muscle function, and reductions in mortality compared to control [37,38]. Explanations as to why losartan could only ameliorate the function of cardiac muscle remain limited, but the primary mechanism could be the significant reduction in afterload seen in the hearts of losartan treated *mdx* mice [38]. Decreased afterload certainly reduces cardiac workload, and this may minimize mechanical injury and subsequent fibrosis to the sensitive cardiomyocytes in *mdx* hearts. Still, the use of losartan as a prophylactic treatment against DMD-related cardiomyopathy seems promising based on these pre-clinical studies and the current clinical availability of losartan (COZAAR™) for its use in hypertension. Future investigation should be directed at evaluating losartan in DMD patients but also, owing to pathway similarity, at comparing the effectiveness of losartan to the many FDA-approved ACEIs.

The definitive mechanism behind reducing cardiomyopathy via ARBs, ACEIs, and/or BBs in DMD patients is not completely established, but reduced aldosterone signaling through ACE inhibition could prevent fibrotic tissue development, as previous use of aldosterone-specific blockers has shown benefit in cases of heart failure [39-42]. Additionally, angiotensin II can directly induce vasoconstriction, pro-fibrotic Smad signaling, pro-fibrotic transforming growth factor beta (TGF-β) production, and the ubiquitin-proteasome pathway that has a role in the proteolysis happening in dystrophic tissues [43-46]. Angiotensin II is also known to enhance NADPH-oxidase activity, which leads to overproduction of superoxide anion and accounts for the oxidative stress in cardiac and skeletal muscle of the mdx mouse [47-52]. ACE inhibition can reduce these adverse effects, and a study with *mdx* mice demonstrated that the ACEI enalapril can prevent angiotensin II dependent stimulation of pro-oxidant and pro-inflammatory pathways [53]. Overall, ACEIs and/or BBs appear to be excellent candidates for DMD therapy based on current clinical availability and demonstrated ability to reduce many of the negative outcomes normally associated with the DMD pathogenic process. Still, a broader investigation regarding the potential prophylactic benefit of ACEIs should be conducted to determine an optimal age of initiation.

#### Phosphodiesterase 5 inhibitors

In the NO-cGMP signaling pathway, nitric oxide synthase (NOS) produces NO to activate soluble guanylyl cyclase (sGC) to synthesize cyclic guanosine monophosphate (cGMP), and cGMP activates protein kinase G to induce vasodilation. The cGMP-specific phosphodiesterases are responsible for cGMP degradation, so vasoconstriction begins as concentrations of cGMP diminish. This pathway is disrupted in dystrophin deficient membranes, as nNOS is absent from the sarcolemma and greatly downregulated, which contributes to the observed functional ischemia [6,19]. Additionally, studies have shown greater cGMP-specific phosphodiesterase 5 (PDE5) activity in *mdx* skeletal muscle samples and decreased cGMP production compared with controls [54,55]. Recently, cGMP-specific PDE5 inhibitors, specifically tadalafil (Cialis™ or Adcirca™) and sildenafil (Viagra™ or Revatio™) have been investigated for their potential in ameliorating the functional ischemia in DMD by increasing intracellular levels of cGMP to prolong vasodilation and increase blood flow to tissues.

Asai *et al*. elegantly showed that tadalafil administration prior to progressive myofiber damage was able to significantly lower the net quantity of myofiber damage in *mdx* mice compared to placebo [24]. Essentially, attenuation of functional ischemia using tadalafil was shown to reduce the extent of contraction-induced damage [24]. Additionally, early treatment utilizing PDE5 inhibitors could have clinical prophylactic benefit, for *mdx* mice treated with tadalafil from conception showed improved histology [24]. Khairallah *et al*. showed that cardiac mRNA expression levels of atrial natriuretic factor (ANF), an early indicator for initiation of cardiomyopathic remodeling, was significantly reduced in *mdx* mice treated with sildenafil [56,57]. This implies that sildenafil is capable of inhibiting the advancement of cardiomyopathic remodeling at early stages of DMD [57]. Sildenafil has also been shown to have positive functional effects in the hearts of *mdx* mice, notably by avoidance of cardiomyocyte damage produced *in vivo* via cardiac workload augmentation and maintenance of an elevated heart rate response for a significantly longer period of time compared with placebo [57]. Interestingly, administration of sildenafil is even capable of reversing cardiac dysfunction in *mdx* mice with established cardiomyopathy [58]. Nevertheless, the target cell and mechanism behind the reversal are still unclear.

Additionally, PDE5 inhibitors have also shown improvement in muscle tissue from other vertebrate models of DMD, namely two dystrophin deficient zebrafish models known as *sapje* and *sapje*-like mutants [59,60]. Dystrophin-null zebrafish treated with aminophylline, a nonselective phosphodiesterase inhibitor, were able to survive significantly longer compared to controls and had restored skeletal muscle structure similar to wild-type zebrafish [61]. Furthermore, analysis of *sapje* mutants treated 1 to 4 days postfertilization with different phosphodiesterase inhibitors revealed that treatment with aminophylline or sildenafil citrate resulted in the lowest percentage of fish showing abnormal muscle structure [61]. These data suggest that aminophylline and sildenafil citrate are capable of preventing the onset of aberrant muscle architecture in dystrophin-null zebrafish. These findings from DMD model zebrafish are analogous to the results seen from *mdx* mice treated with PDE5 inhibitors, and the consistency of these results across several DMD models leaves hope that these compounds could benefit individuals living with DMD. Clinical trials assessing PDE5 inhibitors for DMD patients are currently underway, and future use of tadalafil or sildenafil for these individuals seems promising based on preclinical studies and current clinical availability of these drugs for their use in treating erectile dysfunction and pulmonary hypertension.

### Increased vascular density

#### Vascular endothelial growth factor administration

**Figure 2:**
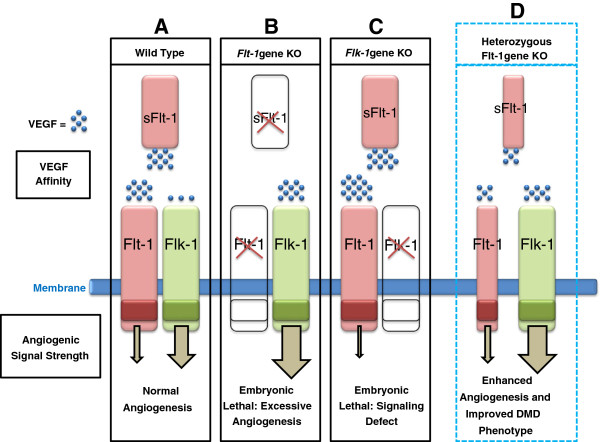
**Flt-1 is a decoy receptor for vascular endothelial growth factor (VEGF) pro-angiogenic signaling.** (**A**) In the wild-type scenario, VEGF induces a pro-angiogenic signal by binding the Flt-1 or Flk-1 receptors [63]. Flt-1 has a higher binding affinity for VEGF but transmits a weaker angiogenic signal compared to Flk-1, which implies that Flt-1 acts a negative regulator of angiogenesis [63]. The soluble form of Flt-1 (sFlt-1) lacks the transmembrane and intracellular signaling domains of Flt-1 and only serves a regulatory role by sequestering VEGF [63]. (**B**) *Flt-1* homozygous knockout (*Flt-1*^−/−^) mice die in the early embryonic stage from endothelial cell overproduction and blood vessel disorganization, indicating that Flt-1 is a decoy regulator for endothelial growth/differentiation [64-66]. (**C**) *Flk-1* homozygous knockout (*Flk-1*^−/−^) mice die in the early embryonic stage from defects in the development of organized blood vessels, indicating that *Flk-1* is a positive regulator for endothelial growth/differentiation [67]. (**D**) Developmental reduction of the Flt-1 receptor through haploinsufficiency of the *Flt-1* gene (*Flt-1*^+/−^) has been shown to increase capillary density in skeletal muscle, and this same phenomenon has been demonstrated in *mdx* mice (*mdx:Flt-1*^+/−^) [79]. The *mdx:Flt-1*^+/−^ mice also showed improved histological and functional parameters normally associated with the Duchenne muscular dystrophy (DMD) pathology [68].

The central paradigm behind the previously discussed PDE5 and ACEI therapies for DMD was that increasing the vasorelaxation capacity of the vasculature would be able to increase perfusion, diminish the effects from functional ischemia, and decrease myocyte damage. However, another technique to increase tissue perfusion would be to increase the density of the underlying vascular architecture that nourishes the skeletal and cardiac muscles. One method of increasing vascular density is to augment angiogenesis, which regulates the production of new vasculatures from the existing framework. The vascular endothelial growth factor (VEGF) family of signal glycoproteins acts as potent promoters of angiogenesis during embryogenesis and postnatal growth. Specifically, the binding of the VEGF-A ligand with the VEGF receptors has been shown to promote vascular permeability and also trigger endothelial cell migration, proliferation, and survival, and the newly formed endothelial cells provide the basic structure of new vasculatures [62]. The dominant VEGF signal molecule for angiogenesis, VEGF-A, mediates its signal through VEGF receptor-1 (VEGFR-1, hereafter Flt-1) and VEGF receptor-2 (VEGFR-2, hereafter Flk-1) [63]. A soluble form of Flt-1 (sFlt-1) also exists, but lacks an intracellular signaling domain and thus only serves in a regulatory capacity by sequestering VEGF-A [63]. Flt-1 and Flk-1 contain an extracellular VEGF-A-binding domain and an intracellular tyrosine kinase domain, and both show expression during the developmental stage and tissue regeneration in hemangioblasts and endothelial cell lineages [63-65]. Flt-1 has a 10 times greater binding affinity for VEGF-A (K_d_ approximately 2 to 10 pM) compared to Flk-1, but the weaker tyrosine kinase domain indicates that angiogenic signal transduction following VEGF-A binding to Flt-1 is comparably weaker than the Flk-1 signal (Figure 2A) [63]. As such, homozygous *Flt-1* gene knockout mice die in the embryonic stage from endothelial cell overproduction and blood vessel disorganization (Figure 2B) [64-66]. Inversely, homozygous *Flk-1* gene knockout mice die from defects in the development of organized blood vessels due to lack of yolk-sac blood island formation during embryogenesis (Figure 2C) [67]. Both the Flt-1 and Flk-1 receptors are needed for normal development, but selective augmentation in VEGF-A concentration should allow for greater binding to the Flk-1 receptor and induce a pro-angiogenic effect that increases capillary density.

Several studies have demonstrated that administration of VEGF using exogenous expression mediated through engineered myoblasts, direct systemic injections, or adeno-associated viral (AAV) vectors are capable of initiating an angiogenic signal in the myocardium and skeletal muscle in both ischemic and non-ischemic conditions [69-72]. However, these same studies do highlight the importance of precisely regulating VEGF delivery quantities for future clinical use, as overadministration has been shown to have deleterious effects in animal models, such as hemangioma formation [70]. Unfortunately, and to the best of our knowledge, functional studies that assess blood flow in DMD model organisms following VEGF-induced angiogenesis have not yet been conducted. However, one study found that four weeks following intramuscular administration of rAAV-VEGF vectors in the bicep and tibialis anterior (TA) muscles of 4-week-old *mdx* mice, the rAAV-VEGF-treated *mdx* mice showed significantly greater forelimb strength compared to pretreatment levels and AAV-LacZ-treated control *mdx* mice [73]. The same study confirmed the feasibility of VEGF-mediated angiogenic induction in *mdx* mice and showed greater capillary density, particularly in the area of regenerating fibers, as well as reduced necrotic fiber area in biceps muscle compared to AAV-LacZ-treated control *mdx* mice [73]. So, although this study did not directly assess reduction of functional ischemia via enhanced vascular density through VEGF-induced angiogenesis, it was able to demonstrate similar outcomes from the PDE5 inhibitor studies, especially the decrease seen in necrotic fiber area and improvement in muscle function.

But apart from the documented pro-angiogenic effect, VEGF delivery also has a powerful pro-myogenic effect. In normal skeletal muscle tissues, VEGF administration induces muscle fiber regeneration and promotes muscle recovery after ischemic and chemical damage [74]. In *in vitro* studies using C2C12 myoblast cell line and primary mouse myoblasts derived from cultured SCs, VEGF was shown to promote growth and protect cells from apoptosis [74]. Similar effects have been documented in dystrophin deficient muscle tissues, where rAAV-VEGF-treated *mdx* mice showed an increase in the area occupied by regenerating fibers and an increased number of activated SCs and developmental myosin-heavy chain-positive fibers in skeletal muscles [73]. *In vivo* transplantation of muscle-derived stem cells (MDSCs) engineered to overexpress VEGF into dystrophic skeletal muscle results in an increase in angiogenesis and endogenous muscle regeneration along with reduction in fibrosis both two and four weeks following transplantation [75]. Thus, the dual functionalities of VEGF, especially the pro-angiogenic and pro-myogenic effects, are capable of improving both the histological and functional parameters normally associated with *mdx* muscle pathophysiology.

These data seem logical because developmentally reducing angiogenesis in *mdx* mice through ablation of matrix metalloproteinase-2 impairs the growth of regenerated myofibers and decreases VEGF expression, further complementing current theories about the close developmental relationship between angiogenesis and myogenesis [76]. But what is the pro-myogenic mechanism of VEGF delivery? SCs are clearly the dominant muscle-specific stem cells utilized for muscle growth, repair, and regeneration, and the number of SCs parallels muscle capillary quantity, largely because SCs reside in a juxtavascular niche [77,78]. In fact, most SCs maintain tight locality to capillaries regardless of character, including quiescent SCs, proliferating SCs (myogenic precursor cells), and differentiating SCs (myocytes), and differentiating myogenin-positive myocytes assessed from DMD muscle biopsies show spatiotemporal association with new capillary growth [78]. A recent *in vitro* study specifically showed that endothelial cells augment myogenic precursor cell growth while differentiating SCs display pro-angiogenic characteristics, thus demonstrating a complementary angio-myogenesis signaling system [78]. Specifically, VEGF stimulated *in vitro* myogenic precursor cell growth, which supports the notion that VEGF is a co-regulatory substance for both angiogenesis and myogenesis [78,79]. Recent work demonstrates that myofibers, SCs or myogenic precursor cells are negative for both Flt-1 and Flk-1, indicating that the effects of VEGF on these cells may be mediated through other VEGF receptors, such as neuropilin-1 (NRP1) and neuropilin-2 (NRP2) [68]. NRP1 and NRP2 can bind to VEGF with a high affinity and act as co-receptors for Flk-1 and Flt-3, respectively [80]. However, other studies have shown that in some developmental cases, the cellular proliferation functions of VEGF via binding to NRP1 are independent of Flk-1 [81]. Thus, the exact mechanism of Flt-1/Flk-1 independent VEGF signaling remains to be elucidated.

An additional element of pro-angiogenic induction is that the expansion of the juxtavascular niche of SCs also serves to increase the basal number of SCs, and this is something not seen in the previously described vasorelaxation strategies [68]. Normally, the successive rounds of regeneration and degeneration seen in *mdx* tissue serves to exhaust the SC pool and reduce the regenerative capacity of the muscle tissue, which decreases SC quantity over time [82,83]. This predetermined decline can be attenuated by improving the regenerative capacity of the muscle through an increase in the number of SCs present, which can be accomplished through expansion of the SC juxtavascular niche, including microcapillaries [78,84,85].

As for the future of VEGF therapies in DMD patients, more data is certainly needed before clinical benefit can be realized. Most important is deciphering effective dosing levels and delivery vehicles, which are both extraordinarily difficult owing to the fact that the body of pharmacology knowledge has shown us that inhibition is easier than introduction. Similarly, there is no agreed upon clinical standard for what constitutes a therapeutic or pathologic increase in the density of the vasculatures. Also, growth factors like VEGF need to be closely monitored due to carcinogenic properties. Similar to the PDE5 inhibitor studies, the effects of VEGF-induced angiogenesis to enhance capillary quantity and mitigate contraction-induced muscle damage via functional ischemia reduction should be further investigated to validate this hypothesis. Moreover, data regarding the functional aspects of the myocardium in dystrophin deficient tissues, the developmental effects of earlier VEGF administration, and the basal quantity of SCs following VEGF-induced angiogenesis in organisms lacking dystrophin all could help ready this therapy for clinical trials in DMD patients.

#### VEGF receptor regulation

A different pro-angiogenic approach that also increases vascular density to ameliorate the functional ischemia in DMD would be to modulate the VEGF receptors. As previously described, Flt-1 acts as a decoy receptor and modulates angiogenesis through its ability to sequester VEGF-A, which reduces signaling through Flk-1. So, although both Flt-1 and Flk-1 are inherently pro-angiogenic, due to the high affinity and low tyrosine kinase activity of Flt-1 over Flk-1 with respect to VEGF-A, Flt-1 acts as a VEGF-A sink preventing Flk-1 access to VEGF-A and thereby functioning as a negative regulator of angiogenesis. This phenomenon has been previously discussed in more detail, and it implies that reduced levels of Flt-1 and/or sFlt-1 could increase the serum concentration of free VEGF-A available to bind Flk-1 and induce a pro-angiogenic response [86].

Interestingly, Flt-1 reduction through haploinsufficiency of *Flt-1* (*Flt-1*^+/−^) produced increased capillary density in skeletal and cardiac muscle compared with control (*Flt-1*^+/+^) in a murine model (Figure 2D) [68]. More significant to this discussion, developmentally reducing Flt-1 through the same genetic method was also shown to further enhance capillary density in the skeletal muscle of *mdx* mice (*mdx:Flt-1*^+/−^) compared to controls (*mdx:Flt-1*^+/+^) [68]. The increased capillary density seen in *mdx:Flt-1*^+/−^ mice serve as a proof of concept that regulating VEGF-A receptors can stimulate a similar pro-angiogenic effect to that seen with direct VEGF-A administration in dystrophin-deficient organisms. More importantly, the changes seen in the *mdx:Flt-1*^+/−^ mice persist into adulthood, yet it remains unclear if this effect must be initiated during developmental stages or if it can be recapitulated in postnatal models.

Analysis of the *mdx:Flt-1*^+/−^ mice with increased capillary density demonstrated improved skeletal muscle histology with a reduction in both myocyte damage and fibers with centrally located nuclei, which strongly suggests that *mdx:Flt-1*^+/−^ fibers display less fiber turnover compared to the *mdx:Flt-1*^+/+^ controls [68]. Additionally, *mdx:Flt-1*^+/−^ fibers show less calcification, fibrosis, and membrane permeability, all of which are downstream effects of dystrophin deficiency [68]. These histological improvements also translated to improved functional parameters such that increased capillary density resulted in increased muscle tissue perfusion and improved skeletal muscle contractile function [68]. Furthermore, Flt-1 has also been assessed in another DMD mouse model: *mdx:utrophin (utrn)*^*−/−*^ mice. These mice, deficient for both dystrophin and the dystrophin-related protein utrophin, display a more severe, progressive form of muscular dystrophy as compared with the *mdx* mice [87,88]. Long term studies using the *mdx:utrn*^*−/−*^*:Flt-1*^*+/−*^ mice with increased capillary density showed significant increases in body mass and survival compared to the *mdx:utrn*^*−/−*^*:Flt-1*^*+/+*^ control mice [68].

Absolute mechanisms that explain the improved phenotype and survival seen in *mdx:Flt-1*^*+/−*^ mice and *mdx:utrn*^*−/−*^*:Flt-1*^*+/−*^ mice remain to be elucidated. But owing to pathway similarity, the explanation is probably similar to descriptions of VEGF-induced angiogenesis in *mdx* mice, namely the close developmental relationship between myogenesis and angiogenesis. Increasing tissue perfusion may compensate for lack of NO-mediated vasodilation, which would attenuate one of the proposed ‘two-hits’ required for myocyte damage. Another theory behind the progressive nature of the DMD pathology is the SC exhaustion model [83]. This theory states that easily damaged DMD myofibers are constantly replaced by endogenous SCs, yet the constant SC cycling leads to rapid shortening of telomerase length and eventual exhaustion of the SC pool [82,83]. Interestingly, *mdx:Flt-1*^*+/−*^ were shown to have developmentally increased numbers of SCs, perhaps mediated through an expanded SC vascular niche. Thus, enhancement in the basal number of SCs could mitigate the accelerated age-related decline seen among SCs from dystrophin-deficient muscle tissue [13].

Overall, Flt-1 is a novel target for pro-angiogenic therapy in DMD. Greater blood perfusion alone seems to compensate for the functional ischemic phenotype in *mdx* mice, but definitive studies showing attenuation of functional ischemia through Flt-1 signal mitigation to reduce the effects of contraction induced damage have not been shown. Flt-1 has also been investigated for its role in cancer where it acts as a positive regulator of the pathological angiogenesis seen with tumor formation [89], which opposes the physiological role of Flt-1 as a negative angiogenic regulator. Thus, numerous small molecules have already been investigated and verified (*in vivo* and *in vitro*) that can antagonize Flt-1 binding to VEGF, reduce angiogenesis, and prevent tumor growth [90]. These same small molecules could be used to selectively block Flt-1 function and promote angiogenesis in dystrophin-deficient tissue. Still, more screening studies could be needed to decipher substances that are viable *in vivo* and can reduce Flt-1 function in order to fully translate the results seen from the developmental studies with *mdx:Flt-1*^*+/−*^ and *mdx:utrn*^*−/−*^*:Flt-1*^*+/−*^ mice using a pharmaceutical agent.

## Conclusions

The role of the vasculature in DMD can no longer be ignored in light of the mounting evidence for its role in the pathogenic process. With this new knowledge in mind and with the dearth of current treatments, this review focused on a variety of new therapeutic options that specifically target these DMD vascular defects, namely attenuation of the functional ischemia (see Table 1 for summary of therapies). One therapy improves systemic vasorelaxation capacity using ACEIs with or without BBs, and this method has shown clinical utility in both preventing and improving the adverse cardiac events normally associated with the DMD phenotype. Treatment using PDE5 inhibitors also improves systemic vasorelaxation capacity, and preclinical evidence from DMD murine models demonstrates the ability of PDE5 inhibitors to prevent skeletal and cardiac muscle damage and even reverse the functional parameters associated with established cardiomyopathy. Both PDE5 and ACEI therapies have a clear practical advantage as they have extensive clinical safety records and many of the drugs are clinically available. There are a wide variety of ACEIs that are FDA approved to treat heart failure and hypertension, including benazepril (Lotensin™), captopril (Capoten™), enalapril (Vasotec™), fosinopril (Monopril™), lisinopril (Prinivil™, Zestril™) moexipril (Univasc™), perindopril (Aceon™), quinapril (Accupril™), ramipril (Altace™), and trandolapril (Mavik™). There are also several PDE5 inhibitors that received FDA approval for treating erectile dysfunction or hypertension, including tadalafil (Cialis™ or Adcirca™), sildenafil (Viagra™ or Revatio™), and vardenafil (Levitra™ or STAXYN™). Additionally, sildenafil has already been extensively studied in a pediatric population and was found to be safe for pulmonary hypertension treatment [91]. Both tadalafil and sildenafil are currently in a phase 1 clinical trial (NCT01580501) that will assess these drugs ability to attenuate functional ischemia in boys with DMD, and other future clinical studies could address the ability of ACEIs to mitigate the same effect.

**Table 1:** Summary of vascular targeted therapies for Duchenne muscular dystrophy

**Treatment**	**Outcome**	**Physiologic effects (mdx mice)**	**Physiologic effects (DMD patients)**	**Potential pharmaceuticals**	**Future directions**
**Angiotensin-converting enzyme (ACE) Inhibitors**	Improved vasorelaxation capacity	Improved myocardial function (prior to onset of cardiomyopathy) [31]. Prevented angiotensin II dependent stimulation of pro-oxidant and pro-inflammatory pathways [51].	Delayed onset and progression of LV dysfunction and lower mortality rates in 9.5 to 13 year olds with normal cardiac functioning [32,33]. Myocardial functional improvements in some cases with established cardiomyopathy [34,36]. Given in combination with BBs, patients with established cardiomyopathy saw positive effect on long term survival [35].	FDA approved ACEIs to treat heart failure and hypertension include: benazepril (Lotensin™), captopril (Capoten™), enalapril (Vasotec™), fosinopril (Monopril™), lisinopril (Prinivil™, Zestril™) moexipril (Univasc™), perindopril (Aceon™), quinapril (Accupril™), ramipril (Altace™), and trandolapril (Mavik™).	Decide effective pharmaceutical agent and use clinical trial to assess potential prophylactic benefit and/or ability to attenuate functional ischemia.
**Phosphodiesterase 5 (PDE5) Inhibitors**	Improved Vasorelaxation Capacity	Decreased myofiber damage after myofiber injury [24]. Improved muscle histology with treatment started at conception [24]. Reduced cardiomyopathy remodeling signals [54,55]. Reversed myocardial dysfunction in models with established cardiomyopathy [56].	N/D^a^	PDE5 inhibitors that are FDA approved for treating erectile dysfunction or hypertension include: tadalafil (Cialis™ or Adcirca™), sildenafil (Viagra™ or Revatio™), and vardenafil (Levitra™ or STAXYN™).	Complete the in-progress phase 1 clinical trial (NCT01580501) assessing the ability of tadalafil and sildenafil to attenuate functional ischemia in boys with DMD.
**Vascular Endothelial Growth Factor (VEGF) Administration**	Increased Vascular Density	Increased forelimb strength and reduced necrotic fiber area [67]. Pro-myogenic effects, including increased regenerating fiber area and number of activated satellite cells in skeletal muscles [67].	N/D^a^	Engineered myoblasts expressing VEGF^b^, VEGF protein systemic injections^b^, adeno-associated viral (AAV) VEGF vectors^b^	Determine best strategy and dosing schedule for delivery, acquire more safety data, and agree on values that constitute therapeutic increases in vascular density. Assess potential prophylactic benefit and/or ability to attenuate functional ischemia in mdx mice.

^a^N/D, not determined. ^b^These are not Food and Drug Administration (FDA) approved and are undergoing pre-clinical investigation in mdx models.

Therapies that enhance the underlying vascular architecture through pro-angiogenic induction include VEGF administration and also VEGF receptor modulation. The pro-angiogenic therapies have shown exciting preclinical proof of concept evidence in DMD murine models, especially the expansion in the basal number of SCs mediated through a larger juxtavascular SC niche and the documented pro-myogenic effects. Still, the pro-angiogenic strategies are in early stages and both methods need definitive means of achieving their desired result and more safety information before clinical trial initiation. In all, the hope is that at least some or combinations of these vascular-targeted therapies will soon have clinical utility and provide current and future human beings living with DMD enhanced control over their own destiny.

## Abbreviations

AAV: Adeno-associated virus; ACE: Angiotensin-converting enzyme; ACEI: Angiotensin-converting enzyme inhibitor; ANF: Atrial natriuretic factor; ARB: Angiotensin receptor blocker; BB: β-blocker; BMD: Becker muscular dystrophy; cGMP: Cyclic guanosine monophosphate; CNS: Central nervous system; DAPC: Dystrophin-associated glycoprotein complex; DMD: Duchenne muscular dystrophy; EDHF: Endothelium-derived hyperpolarizing factor; LVEF: Left ventricular ejection fraction; MDSCs: Muscle-derived stem cells; nNOS: Neuronal nitric oxide synthase; NO: Nitric oxide; NOS: Nitric oxide synthase; NRP1: Neuropilin-1; NRP2: Neuropilin-2; PDE5: Phosphodiesterase 5; sGC: Soluble guanylyl cyclase; SC: Satellite cell; rAAV: Recombinant adeno-associated virus; TA: tibialis anterior; TGF- β: Transforming growth factor-β; VEGF: Vascular endothelial growth factor; VEGFR: Vascular endothelial growth factor receptor.

## Competing interests

The authors have no financial competing interests.

## Authors’ contributions

JPE completed the literature review, developed the figures, and prepared the review. MV revised the manuscript. AA advised the literature review process and revised the manuscript. All authors read and approved the final manuscript.

## Authors’ information

JPE is a staff researcher in the laboratory of Dr. Atsushi Asakura at the University of Minnesota Stem Cell Institute.

MV is an MD/PhD candidate through the Medical Scientist Training Program at the University of Minnesota Medical School.

AA is an Assistant Professor of Neurology and a faculty member of the Stem Cell Institute in the University of Minnesota Medical School. He also belongs to the Paul & Sheila Wellstone Muscular Dystrophy Center in the University of Minnesota Medical School.
